# Can language use in social media help in the treatment of severe mental illness?

**DOI:** 10.46439/psychiatry.1.001

**Published:** 2021

**Authors:** Deanna L. Kelly, Max Spaderna, Vedrana Hodzic, Glen Coppersmith, Shuo Chen, Philip Resnik

**Affiliations:** 1 University of Maryland Baltimore, School of Medicine, Baltimore, MD, USA; 2 Qntfy, USA; 3 University of Maryland College Park, MD, USA

Nationally, patients experience multiple barriers to receiving mental health care. In many parts of the US, access to mental health providers is limited [1]. For many patients, getting an appointment with a psychiatrist is difficult [2] and often takes weeks [3]. When patients are able to schedule appointments with a psychiatrist, the visits are usually short and aimed mostly at prescribing medications [4]. For patients with serious conditions like schizophrenia or major depression, the consequences of unattended emergence or worsening of symptoms during those time intervals can be severe. Thus, technology to provide clinicians with additional information between patient visits—what Coppersmith and colleagues have dubbed the “clinical whitespace”—could be a valuable tool. Technological tools cannot, and should not, replace the all-important physician-patient relationship, but they may help physicians utilize all the available data to provide the best possible care.

The language of patients offers a key opportunity to gain insight into their condition between appointments. A growing body of research indicates that language use contains valuable evidence for the evaluation and monitoring of mental health symptoms [5–7]. For example, some aspects of language use commonly seen in patients diagnosed with schizophrenia [8–14] can help predict which high-risk patients are likely to develop psychosis [15]. Aspects of language use have also been related to illness and illness severity in patients with depression [16–19].

The widespread use of social media provides an opportunity to access everyday language in people with schizophrenia and depression. Notwithstanding the continuing evolution of new platforms, Facebook remains a prime example: recent data shows that 69% of all U.S. adults are Facebook users with 74% of U.S. adults visiting the site at least once a day [20]. Those with severe mental illness also utilize social media frequently, with over 50% of people with schizophrenia using social media in the past week [21]. Furthermore, studies indicate that people are frequently willing to consent to use their private social media data for research [22–24], and are open to the idea of opt-in use of social media under clinical supervision [24,25]. This suggests that patient acceptance would not be a significant barrier for social media-based technological tools assisting their clinicians in their care.

Taking advantage of this data requires connecting evidence from language use in social media to mental health conditions and biomedical outcomes. Growing evidence shows that it is possible to connect the dots in this way. De Choudhury and colleagues [26,27] and Coppersmith and colleagues [28–30] pioneered early work using social media data to evaluate depression and other conditions, with an energetic community of researchers further developing methods of social media data analysis for depressive disorders and schizophrenia using Twitter [31–35], detection of depression using Facebook data [36–40], and identification of crisis or self-harm risk via social media activity on multiple platforms [30,41,42]. Increasingly comprehensive reviews on this topic have begun to emerge [43–49].

Some of our own recent work has also contributed to understanding the potential of social media data in mental health care, by focusing on clinically valid evidence about psychiatric symptoms in social media posts–that is, the connection between evidence available in social media and standard clinical measures used in traditional in-person assessments. This complements a larger body of work in which proxy variables, such as an individual posting in a relevant mental health forum or self-reporting a diagnosis, are used in lieu of actual clinical diagnoses and ratings, the latter often being much more difficult to obtain; this is important given an increased understanding of the limitations from this proxy approach [49,50]. In a pilot study [51], we had a group of psychiatrists and other clinicians provide symptom ratings for individuals based only on reading their Facebook posts, and these were compared with independent clinical ratings of the same individuals generated via in-person interviews with trained clinicians. The trends in our between-groups comparison indicate that the posts do indeed contain predictive signal about standard assessment constructs, an essential step toward technologies that can present useful information to clinicians. Prediction of outcomes has shown significant promise in work by Coppersmith et al. [30] predicting suicide attempts from Facebook and Twitter data, Birnbaum et al. [52] predicting relapse and hospitalizations in first-episode-psychosis patients using Facebook data contributed by patients, and Corcoran et al. [15], predicting transitions to psychosis using language in people at high risk for psychosis.

Moving from this promising start to deployed technologies that harness social media signal for characterizing illness will require further research, careful testing, and close engagement between technologists and clinicians. It will also require careful consideration of ethical issues and barriers to acceptance, particularly in light of concerns about privacy and use of social media content by tech giants and those to whom they provide users’ data [53–55], as well as fairness and bias in automated systems [56,57]. Significant energy is being devoted to sorting out these issues and developing appropriate guidelines [49,58], and secure data enclaves have begun to address data privacy concerns by bringing researchers to the data rather than vice versa [59]. It is crucial to remember that proper ethical consideration involves both risks and benefits. We have argued in previous work [60] that although the lack of careful ethical review for technological work on mental health is obviously unacceptable, “rejecting technology-driven research … out of anxiety regarding potential ethical concerns, without carefully considering relevant guidelines or standards in this area, is, in itself, a breach in ethics. It is discriminatory–a condition-based form of bias analogous to the neglect of women in cardiology research … or underrepresentation of minority populations in clinical trials”. With that in mind, a related and important consideration for using this type of technology in the future to assist mental health care is the extent of availability of social media data. Despite increasingly wide access, not all populations have access to the internet in the same capacity, and even when it is available, different populations may use technology in different ways. Minority groups, elderly, illiterate people, homeless, and incarcerated populations may have less opportunity to provide this type of information and data, and this issue will require careful attention in the development and introduction of new technologies supporting complementary care.

Fully consented opt-in data donation has emerged as one valuable tool for balancing the data requirements of machine learning systems with the ethical acquisition and use of individuals’ clinical and social media data [22,30]. The work by Coppersmith et al. [30] on the prediction of suicide risk makes use of data from OurDataHelps.org, a platform for the consented donation of online data that as of this writing (January 2021) has collected self-report mental health data and social media content from more than 4000 individuals. Using a variant of the OurDataHelps platform at UMD.OurDataHelps. org, our own similar data collection efforts in connection with schizophrenia and depression have enrolled more than 4500 data donors to date, where 82% of individuals who began the process have completed enrollment, not withdrawn from research, completed mental health questionnaires, and contributed social media data.^1^

## Conclusion

From the provider perspective, technological analysis of language in social media offers the potential for a more comprehensive picture of the patient, foregrounding clinically valid signals about patients’ mental state and experiences by using social media to provide a window into the clinical whitespace, in order to facilitate management and intervention for mental health conditions [61,62]. New information that was previously unavailable to physicians can provide a window into daily life by using language in the virtual world to aid in decision making, dosing adjustments, medication changes, identification of symptom changes, and, more generally, understanding the patient’s lived experience–providing essential information in real time at the point when patient worsening or relapse may become evident.

From the patient perspective, the same technologies offer the potential for better understanding of their own mental state, more informed decision making, and more effective partnership with their providers. Evidence suggests that people who identify as having mental illness are receptive to the introduction of technologies of this kind [24,25], including delivery of mental health programs through social media [63].

Achieving this potential requires careful consideration of clinical, ethical, and practical issues, and above all it requires an increased level of understanding and partnership between technologists and clinicians. But if done well, technologically supported analysis of social media is a powerful new source of information that can be developed and used ethically and effectively to improve outcomes and the lives of the patients that we treat.

## Footnotes

1 Our IRB-approved data collection includes secure website enrollment, participant consent, and permission to access and collect data by a third-party technology solutions company with appropriate steps to ensure the data is anonymous and secure. Similar approaches can be found elsewhere, as well (e.g. [41]).
